# Insights into the Host-Pathogen Interaction Pathways through RNA-Seq Analysis of *Lens culinaris* Medik. in Response to *Rhizoctonia bataticola* Infection

**DOI:** 10.3390/genes13010090

**Published:** 2021-12-29

**Authors:** Gyan P. Mishra, Muraleedhar S. Aski, Tejas Bosamia, Shiksha Chaurasia, Dwijesh Chandra Mishra, Jyotika Bhati, Atul Kumar, Shaily Javeria, Kuldeep Tripathi, Manju Kohli, Ranjeet Ranjan Kumar, Amit Kumar Singh, Jyoti Devi, Shiv Kumar, Harsh Kumar Dikshit

**Affiliations:** 1 Division of Genetics, Indian Agricultural Research Institute, New Delhi 110012, India; gyan.gene@gmail.com (G.P.M.); murali2416@gmail.com (M.S.A.); chaurasia.shiksha785@gmail.com (S.C.); manjukohli1996@gmail.com (M.K.); 2 Plant Omics Division, Central Salt and Marine Chemicals Research Institute, Bhavnagar 364002, India; tejasbosamia@gmail.com; 3 Agricultural Bioinformatics, Indian Agricultural Statistics Research Institute, New Delhi 110012, India; dwij.mishra@gmail.com (D.C.M.); singh.jyotika@gmail.com (J.B.); 4 Division of Seed Science and Technology, Indian Agricultural Research Institute, New Delhi 110012, India; atulpathiari@gmail.com (A.K.); shailyjaveria@gmail.com (S.J.); 5 Germplasm Evaluation Division, National Bureau of Plant Genetic Resources, New Delhi 110012, India; kdtripathi89@gmail.com; 6 Division of Biochemistry, Indian Agricultural Research Institute, New Delhi 110012, India; ranjeetranjaniari@gmail.com; 7 Division of Genomic Resources, National Bureau of Plant Genetic Resources, New Delhi 110012, India; amitsinghbiotech@gmail.com; 8 Division of Crop Improvement, Indian Institute of Vegetable Research, Varanasi 221305, India; jyoti17iivr@gmail.com; 9 Biodiversity and Integrated Gene Management Program, International Center for Agricultural Research in the Dry Areas, Avenue HafianeCherkaoui, Rabat 10112, Morocco

**Keywords:** *Lens culinaris*, *Rhizoctonia bataticola*, RNA-Seq, transcription factors, plant defense, signal pathway

## Abstract

Dry root rot (*Rhizoctonia bataticola*) is an important disease of lentils (*Lens culinaris* Medik.).To gain an insight into the molecular aspects of host-pathogen interactions, the RNA-seq approach was used in lentils following inoculation with *R.* *bataticola*. The RNA-Seq has generated >450 million high-quality reads (HQRs) and nearly 96.97% were properly aligned to the reference genome. Very high similarity in FPKM (fragments per kilobase of exon per million mapped fragments) values (*R* > 0.9) among biological replicates showed the consistency of the RNA-Seq results. The study revealed various DEGs (differentially expressed genes) that were associated with changes in phenolic compounds, transcription factors (TFs), antioxidants, receptor kinases, hormone signals which corresponded to the cell wall modification enzymes, defense-related metabolites, and jasmonic acid (JA)/ethylene (ET) pathways. Gene ontology (GO) categorization also showed similar kinds of significantly enriched similar GO terms. Interestingly, of the total unigenes (42,606), 12,648 got assembled and showed significant hit with *Rhizoctonia* species. String analysis also revealed the role of various disease responsive proteins viz., LRR family proteins, LRR-RLKs, protein kinases, etc. in the host-pathogen interaction. Insilico validation analysis was performed using Genevestigator^®^ and DEGs belonging to six major defense-response groups viz., defense-related enzymes, disease responsive genes, hormones, kinases, PR (pathogenesis related) proteins, and TFs were validated. For the first time some key miRNA targets viz. miR156, miR159, miR167, miR169, and miR482 were identified from the studied transcriptome, which may have some vital role in *Rhizoctonia*-based responses in lentils. The study has revealed the molecular mechanisms of the lentil/*R.* *bataticola* interactions and also provided a theoretical approach for the development of lentil genotypes resistant to *R.* *bataticola*.

## 1. Introduction

Lentil (*L. culinaris* Medik.) is an important edible legume and annual self-pollinating crop with an average genome size of 4 Gbp [1,2]. Lentil which is also known as red dhal or Masur provides an inexpensive source of nutrition including carbohydrates, proteins, dietary fibers, vitamins, and micronutrients to a large section of the poor population that are residing in developing countries [3,4,5,6]. Lentil is also a rich source of non-nutritional components such as tannins and phytic acid which plays a key role in imparting defense against fungus, insects, and parasites [7,8]. Globally, the yield of lentils was nearly 5.73 million tons (from 4.8 m ha) in 2019 with Canada being the largest producer (2.17 mt from 1.49 m ha), followed by India (1.23 mt from 1.36 mha), and Australia (0.53 mt from 0.36 m ha). The overall productivity of India is very poor (901 kg/ha) when compared with that of world productivity (1194.6 kg/ha) or that of Canada (1455.7 kg/ha) or Australia (1482.2 kg/ha) [9]. In India, Madhya Pradesh and Uttar Pradesh are the largest producers with more than 70% of the total production followed by West Bengal and Bihar.

Dry root rot, caused by *R.* *bataticola* (Taub.) Butler. (Synonym: *Macrophomina phaseolina* (Maubl.) Ashby.), is a major emerging seedling disease that forms microsclerotia in many Leguminosae crops including lentils under dry and humid climatic conditions. This fungus can live up to 15 years in extreme higher temperatures (30–35 °C), diverse pH conditions, low soil moisture, salt, and drought situations [10,11,12]. *R.* *bataticola* is a soil-inhabiting pathogen and was first reported from Pakistan [13]. It infects more than 500 cultivated and wild plant species including maize [14], mungbean [15], sesame [16], lentils [17], etc. The disease symptoms include the sudden drooping of top leaves, drying of roots, and there is development of black sclerotial bodies on the root surface which ultimately results in stunted plant growth [17].

The increasing incidence of dry root rot has recently been reported from different parts of the world, causing severe yield losses, especially under high temperature and soil-moisture deficit stress conditions [18]. In 2002, this fungus reportedly caused yield losses up to $173.80 million in soybean in the USA, while in Bangladesh, this pathogen has caused nearly a 30% fiber yield reduction in jute [19]. However, no such accurate yield loss report is available for lentils. Till now, there is an unavailability of any effective chemical control or any absolute disease-resistant genotypes that are known for the management of this deadly disease. Despite various efforts, little is known about the molecular mechanism of the host’s (lentil) reaction when infected by the pathogen (*R.* *bataticola*). Thus, for the inclusive understanding of the host-pathogen interactions, it is required to have comprehensive information about the differential gene expression details during infection in both the pathogen and the host so that the detailed infection mechanism can be thoroughly understood.

Advancement in the understanding of the host-pathogen interactions has been accredited by the availability of plant and pathogen genome sequences and the generation of next-generation sequencing (NGS) tools. Thus, comparative RNA-seq analysis has become a comprehensive approach for the identification of DEGs under infected and control conditions that help in the identification of the precise gene networks that are operating under infection, especially in the susceptible genotype [20,21]. This method has been widely applied to identify the plant-pathogen interactions in several crops such as rice [22], peanut [23], mungbean [24], and lentil [25,26].

A class of small non-coding RNAs (~21-nucleotide) is called micro-RNA (miRNAs) which are known to control the post-transcriptional expression of mRNAs in several species [27]. miRNAs function by interacting with the target mRNAs when loaded into the Ago proteins [28] and, thereby, prevent the gene expression by mRNA repression and destabilization [29]. With the advent of better bioinformatics tools, it is now possible to identify the putative miRNAs from an RNA-Seq dataset by identifying the miRNA binding sites. Thus, the precise identification of miRNA target sites from the lentil RNA-seq dataset will help in the better understanding of the *Rhizoctonia* disease response pathways.

The defense mechanisms in the plants are being regulated in different ways and one such mechanism is effector-triggered immunity (ETI) which is caused by the effector identification by the R-proteins or disease resistance proteins of the plant. Many R-proteins are of NBS-LRR (nucleotide-binding site leucine-rich repeat)-type proteins [30]. The defense response by the plant is also regulated by several interconnected pathways which get triggered when PAMPs (pathogen-associated molecular patterns) are recognized by the transmembrane pattern recognition receptors (PRRs); which are known as PAMP-triggered immunity (PTI). Pathogens can overcome the PTI by way of secreting small molecules of pathogenic effectors into the host [23,24].

During host-pathogen interaction, a number of DEGs that are associated with the various signaling cascades have been reported causing formation of various defense-related proteins (LRR family proteins, LRR-RLKs, protein kinases, etc.), TFs (transcription factors), antioxidative enzymes, cell-wall modifying enzymes, phenolic compounds, hormone signals JA/ET pathways, fungal elicitors recognition, hypersensitive response (HR), systemic acquired resistance (SAR), miRNAs (viz. miR156, miR159, miR167, miR169, miR482), and several secondary metabolites. Thus, RNA-Seq analysis can be very effectively used as a tool to find the genome-wide transcriptional changes to the genes and proteins functions during plant-pathogen interaction [23,24,31]. Now, reports are appearing that demonstrate the differential expression of various genes as a result of the host-pathogen interactions in different crops including *Ascochyta lentis* infection (in lentil) [31].

However, till now there is no report about the comprehensive understanding of the defense response in lentil plants to the dry root rot infection. An oriented RNA-Seq approach during lentil-*Rhizoctonia* infection would help in unfolding the molecular response of lentils to the deadly *Rhizoctonia* root rot. With this backdrop, this study was aimed to find the host (lentil)-pathogen (*R.* *bataticola*) interaction network using the RNA-Seq approach for a detailed understanding of the disease response at the molecular level.

## 2. Materials and Methods

### 2.1. Plant Materials and Fungal Inoculation

**Figure 1:**
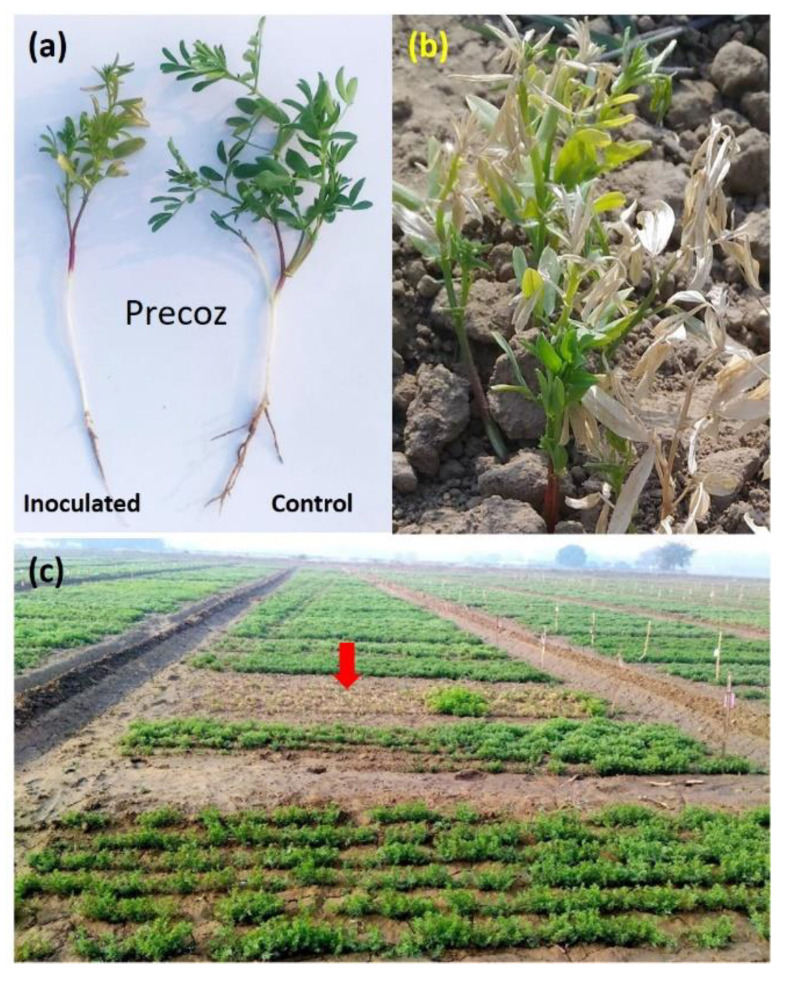
Lentil genotype Precoz that was infected with *R.* *bataticola*. (**a**) Infected and control seedling, (**b**) Infected seedlings showing wilting symptoms, (**c**) Infection under field conditions where red arrow indicates a dried patch of lentil crop due to the *R. bataticola* infection under field conditions.

A dry root rot-susceptible lentil genotype, Precoz was used for the RNA-Seq analysis. Precoz was identified as the susceptible genotype to dry root rot infection at IARI, New Delhi during the year 2017–2018 under field conditions (Figure 1) and was subsequently reconfirmed for its susceptibility under partially control glasshouse conditions by infecting it with the *R.* *bataticola*. The infected plant samples were collected from the field of Indian Agriculture Research Institute, Pusa New Delhi (Latitude: 28°38′30.5″, Longitude: 77°09′58.2″ and Altitude: 228 m AMSL), and a homogeneous pure culture was isolated on PDA (potato dextrose agar) media from surface-sterilized necrotrophic root tissues under lab conditions. Pure culture colonies were grown on PDA at 25 ± 2 °C under 12 h light conditions for 7 days for culture morphology studies [32].

The morphological identification was confirmed by the appearance of colony as well as observed with an Olympus compound microscope (20× magnification) after slide mounting was done using lactophenol. This was again confirmed at the genomic level by the isolation of DNA from the fungal mycelia (and sclerotia) and PCR-based amplification of the internal transcribed spacer (ITS) region of ribosomal DNA using universal primers ITS1 and ITS4 [33,34] and sequencing of the product, which was then submitted to the NCBI (Accession No. OL304938).

Lentil seedlings that were 30-days old of the susceptible genotype Precoz were used for the infection were grown in 15 cm (diameter) pots (three seeds per pot) containing grow-media consisting of coco peat: vermiculite: sand in 1:2:1 ratio. Seedling inoculation was performed using *R. bataticola*, which was mass multiplied on autoclaved sorghum seeds (presoaked in water) and mixed in the soil [35]. For the control plants, only sorghum seeds (non-colonized) were mixed in a similar fashion in the growing media. Afterward, the pots were covered with a moistened plastic bag for 24 h. The symptoms appeared 4 days after inoculation while the control remained healthy. The plants were grown under the partially controlled glasshouse conditions (28 °C to 18 °C during day and night, 14:10 h as light:dark period, 70–80% RH) of the National Phytotron Facility at IARI, New Delhi, and were watered at 3–4 days interval.

For RNA-seq analysis, the infected samples (after 72 h of inoculation) and control samples (mock-inoculated) were taken out from the pots and the roots were washed with distilled water to remove the soil debris. The roots were then cut into small pieces and dipped in the RNA later solution and immediately stored at −80 °C before RNA extraction. A total of four samples were used for the study which consisted of the infected (*R.* *bataticola* inoculated) and the control samples in two replications each.

### 2.2. RNA Extraction, cDNA Library Construction, and Illumina RNA-Sequencing

Total RNA was extracted from the frozen root samples of the control and the infected lentil plant using the RNeasy plant mini kit (Qiagen, Hilden, Germany) following the manufacturer’s protocol. Genomic DNA was then removed from the samples by giving DNAase I (DNase I, Thermo, Waltham, MA, USA) treatment twice during and after RNA extraction, and the RNA was dissolved in nuclease-free water. The RNA quality and integrity were measured using a Bioanalyzer 2100 RNA 6000 Nano Kit (Agilent Technologies, Santa Clara, CA, USA) and on 2% agarose gel, respectively. Then, an equal amount (1.5 µg) of RNA samples (OD 260/280: 2.0~2.1, OD 260/230: ≥2.0~2.3, RIN: ≥7.0) were taken from two biological replicates (from each control and infected root samples) for the construction of four cDNA libraries using a TruSeq mRNA Library Prep kit (Illumina Inc., San Diego, CA, USA). The cDNA libraries were used to generate 100 bp paired-end reads (100 × 2 = 200 bp) using Illumina Hiseq 2500 at AgriGenome Labs Pvt Ltd., Hyderabad, Telangana, India. The samples were labeled as Precoz-R-Root-B1 and Precoz-R-Root-B2 (mock-inoculated or control lentil roots in two replications), Precoz-S-Root-A1 and Precoz-S-Root-A2 (*R.* *bataticola* inoculated lentil roots in two replications). The Illumina sequencing data of these four samples (PE libraries) were then deposited in the United States National Center for Biotechnology Information (NCBI) SRA database (Accession number PRJNA779234).

### 2.3. De Novo Transcriptome Assembly

The raw reads were first analyzed using FastQC (www.bioinformatics.babraham.ac.uk/projects/fastqc, accessed on 21 February 2021), and then the adapters and low-quality bases (Phred score ≤ 30) were trimmed, followed by adapter trimming using AdapterRemoval2 (ver. 2.2.0; https://github.com/MikkelSchubert/adapterremoval, accessed on 21 February 2021). The rRNAs were removed through bowtie2 and then the GC content, Q 30, and clean reads were determined. Afterward, the high-quality clean reads were assembled using Trinity software ver. 2.4.0 (http://trinityrnaseq.sourceforge.net, accessed on 21 February 2021) [36] using the default parameters using a short read assembling program.

### 2.4. Transcriptome Annotation and Gene Ontology (GO)

The filtered reads from each sample were then aligned to the assembled unigenes (Transcript length ≥200 bp) using Bowtie2 ver. 2.2.2.9 (http://Bowtie2-bio.sourceforge.net/index.shtml, accessed on 21 February 2021) and were used for the estimation of transcript expression and their downstream annotations. Before differential expression analysis of the genes, the read counts from each sample were normalized. FPKM was used to find the expression level of the individual transcripts. The assembled unigenes were annotated using Uniprot Plant Database using BLASTX, organism annotation, gene, and protein annotation to the matched transcript. The unigenes, which does not hit earlier, was considered for a homology search with Uniprot plant protein database using Diamond (BLASTX) program ver. 0.9.3.104 (https://github.com/bbuchfink/diamond.git, accessed on 21 February 2021). The GO annotations of the assembled unigenes were obtained using the BLAST2GO (https://www.blast2go.com, accessed on 21 February 2021) [37].

### 2.5. Differential Gene Expression Analysis

Gene expression analysis was performed based on the count of each transcript expression level in the control and infected samples using EdgeR (http://www.bioconductor.org/packages/2.12/bioc/html/edgeR.html, accessed on 14 August 2021) software [38]. The relative expression of the genes having a false discovery rate (FDR) ≤ 0.05 and Log2 Fold Change +2/−2 were used as a threshold for identifying DEGs during up-or down-regulation. The gene expression values that were normalized using FPKM were used for the calculation of fold change in the expression levels using Cuffdiff (http://cole-trapnelllab.github.io/cufflinks/cuffdiff/, accessed on 14 August 2021).

### 2.6. GO-ENRICHMENT and Pathway Analysis

GO-enrichment analysis was performed using ‘goseq’ of ‘R package’ (https://bioconductor.org/packages/release/bioc/html/goseq.html, accessed on 14 August 2021) and analysis was executed independently for up-and down-regulated genes. GO-enriched terms were then visualized as a scatterplot using Revigo (http://revigo.irb.h, accessed on 14 August 2021) [39]. Metabolic pathway analysis of the assembled unigenes was performed using the information that was available in the KEGG (Kyoto Encyclopedia of Genes and Genomes) Automatic Annotation Server (KAAS) (http://www.genome.jp/kegg/, accessed on 14 August 2021) database [40].

### 2.7. PHI-Base Analysis

A homology search using BLASTX [E value ≤ 0.0001] was performed for all the DEGs against the protein sequences that were downloaded from the latest release of PHIbase (https://raw.githubusercontent.com/PHIbase/data/master/releases/phi-base_fasta_v4-6_2018-12-05.fas, accessed on 21 February 2021). The analysis was aimed to find the cross-kingdom comparative network for pathogenicity, virulence, and effector genes and also to identify the key biotic stress-associated targets during lentil-*Rhizoctonia* infection [41,42]. 

### 2.8. Identification of miRNA and miRNA Target Sites

The sequences were searched against the mirBase database for the identification of precursor miRNA sequences, which were scanned for hairpin-like secondary structures using the miRNA identification pipeline of the C-mii software [43]. Only candidate sequences fitting the following criteria were considered as putative miRNAs viz., the length of the predicted mature miRNAs should be in the range of 18–24 nucleotides; a maximum of two mismatches compared with known rice mature miRNAs should be allowed; the mature miRNA should be localized in only one arm within the predicted stem-loop structure; no more than five mismatches should be allowed between the miRNA sequence and the guide miRNA sequence in the stem-loop structure; and miRNAs should have high A  +  U content (30–70%), and minimal folding free energy (MFE) and minimal free energy index (MFEI) value of the secondary structure should be highly negative.

Further, to understand the biological functions of miRNAs, putative target genes were identified from the lentil RNA-Seq data using psRNATarget (https://www.zhaolab.org/psRNATarget/, accessed on 14 August 2021) against the available *Arabidopsis thaliana* annotated data using matching scoring schema [44]. Based on the score, a miRNA-target network was built among miRNA, and their target genes using Cytoscape software [45].

### 2.9. Protein-Protein Interaction (PPI) Network

To understand the PPI network of the miRNA-associated targets, a String network analysis was performed. The selected target lists were first uploaded into the STRING database (https://string-db.org/, accessed on 14 August 2021) [46] and then the MCODE plugin of Cytoscape v3.7.0 [45] was performed for the identification of highly interconnected proteins. The predicted PPI networks were based on the sequence similarity of the model crop *A.* *thaliana*.

### 2.10. InSilico Validation Analysis Using Genevestigator^®^

The DEGs were first shortlisted depending upon the stress-related GO terms and were further narrowed down based upon their FDR value (FDR < 0.05). The peptide sequences of the shortlisted DEGs were extracted from the Uniprot database and then BLASTP was run. Finally, 270 unique target accession numbers were submitted as input in the Genevestigator software [47,48] to find the associated reference genes having the highest stability of expression against the selected organism i.e., *A.* *thaliana* taking AGRONOMICS whole-genome tiling array platform. A hierarchical clustering tool was also used to group the target genes having similar profiles across the above condition. We have also obtained the log2 ratio change value for the identified genes and the top 15 perturbations were presented as a heat map.

## 3. Results

### 3.1. Fungus Confirmation and Inoculation

Slide mounting has shown the following morphological features of the fungus viz., cylindrical hyphae with branching at 90° angle having septum next to the branching point (Figure S1). This is similar to that which is described for *Rhizoctonia solani* [49]. The sequence was then deposited in GenBank (Accession No. OL304938.1) which has shown to have a 99.67% similarity with *M.* *phaseolina* isolate MpOc01 (*R. bataticola*, Accession No. KT768128.1) at 100% query cover.

### 3.2. RNA Sequencing and De Novo Transcriptome Assembly 

The RNA was extracted from the root tissues of *L.* *culinaris* plants from both the control (mock-inoculated) and the infected (*R.* *bataticola* -inoculated) plants and transcriptional changes were analyzed during fungus infection over the control using Illumina Hiseq 2500 platform. The control and inoculated samples were designated as Precoz-Root-B1R & Precoz-Root-B2R and Precoz-Root-A1S & Precoz-Root-A2S, respectively. The RNA-Seq generated nearly 452 million reads from all four sample combinations with an average GC content of 43.48%. The raw read sequences ranged from nearly 114.38 million to 119.7 million (for control: Precoz-Root-B1R & Precoz-Root-B2R) and 107.46 million to 110.53 million (for inoculated: Precoz-Root-A1S & Precoz-Root-A2S), respectively. After passing the low-quality raw reads and trimmed adapters sequences and rRNA sequences, about 450 million (99.65%) HQRs were obtained having a Phred score ≥30 (Table 1). The clean reads ranged from nearly 113.8 million to 119.3 million for resistant (control) and 107,170,090 to 110,235,896 for susceptible plants with an average Q30 quality score of 93.96% and average GC content of 43.48%. The summary of the various parameters of the RNA-Seq data of the inoculated and control samples are given in Table 1. 

The HQRs from the four samples were then aligned to assemble using the Trinity program with default options. Of all the reads, 96.82% to 97.06% of the reads from each sample could properly be aligned back to the assembled unigenes, indicating that the assembly showed high-quality read data (Table 1). The total number of paired-end (PE) reads that were obtained in the control sample ranged from 56.90 million to 59.67 million; while in the inoculated sample this ranged from 53.58 million to 55.11 million. All the assembled transcripts were ≥200 bp long with a GC content from 42.88 to 44.01%. The assembly generated 127,577 transcripts with the longest transcript being 16,804 bp, and the average transcript length was 7974 bp. A total of 115,475 unigenes were generated and the length of the unigenes ranged from 904 bp to 15,536 bp with an average value of 7217 bp (Table S1; Figure S2).

### 3.3. Differential Gene Expression Analysis

Using the denovo transcriptome assembled, first, the total number of genes that were expressed in the control (mock-inoculated) and the susceptible (*R.* *bataticola*-inoculated) samples were identified, and then the DEGs were identified using the edgeR program. A total of 42,479 unigenes were considered for differential gene expression analysis between the control and infected samples (Precoz-S-Root-A vs. Precoz-R-Root-B) at the threshold level (FDR ≤ 0.05; Log2FC +2/−2) (Table S1). A total of 6075 DEGs were identified, of which 2838 were up-regulated and 3237 as down-regulated when a comparison was made between the control and inoculated libraries (Figure S3; Table S1). The DEGs in different combinations were visualized as Volcano and MA plots (Figure S3). The consistency among the samples was verified by performing the correlation analyses that was based on the FPKM values. The high similarity (Figure S2; Table S2; *R* > 0.9) among the FPKM values of the two biological replicates of the control and fungal-infected samples demonstrated that the RNA-Seq results were very much consistent. 

### 3.4. Transcriptome Annotation 

To identify the function of *L.* *culinaris* unigenes, the assembled unigenes were annotated using Uniprot Plant Database. The assembled unigenes were first searched against the protein sequences of *Rhizoctonia* species that were available in the UniProt database. Those unigenes which did not hit earlier were considered for a homology search with Uniprot plant protein database using BLASTX program. Among the total unigenes (42,606) which were considered for annotation, using UniProt Plant Database, 12,648 assembled unigenes that had a significant hit with *Rhizoctonia* species and 25,789 unigenes had at least one significant hit with UniProt Plant Protein Database could be identified (Table 2). The complete annotation summary is provided in Table S3.

Compared with the other species databases, unigene sequences exhibited the most similar BLASTx matches to the gene sequences from *Medicago truncatula* (11828) followed by *Cicer arietinum* (6686), *Trifolium pratense* (4925), *Thanatephorus cucumeris* (strain AG1-IB/isolate 7/3/14) (*R.* *solani*) (3843), *R.* *solani* 123E (3391), *R.* *solani* (2750) and *R.* *solani* AG-8 WAC10335 (1217), and *R.* *solani* AG-3 Rhs1AP (841) (Figure S4). The top BLASTX hit of each unigene and organism name were extracted and the top 25 organisms are shown in Figure S4. The annotation details of the total DEGs with that of *Rhizoctonia* are presented in Table S4.

### 3.5. GO-Enrichment and Pathway Analysis 

**Figure 2:**
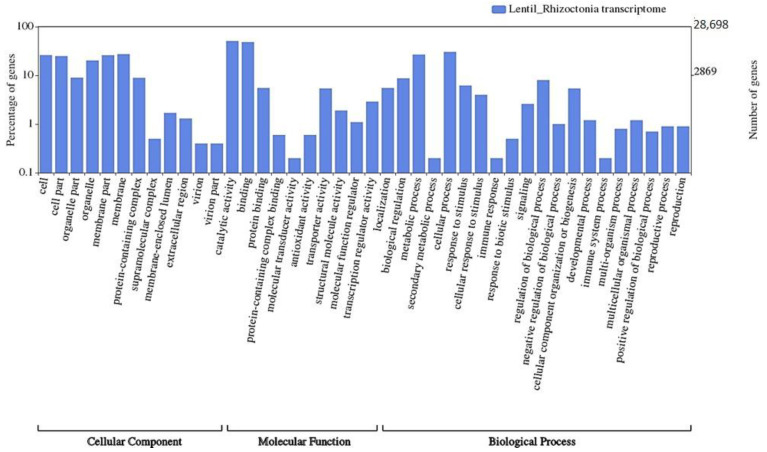
Functional annotation of unigenes that are based on GO categorization of lentil and *Rhizoctonia* transcriptome. These GO terms are classified into three categories (cellular component, molecular function, and biological processes).

The GO terms for unigenes were extracted and were functionally assigned to GO terms and successfully classified into three major GO categories using the Blast2GO program (http://www.blast2go.com/, accessed on 21 February 2021) viz., Biological Process (1296), Cellular Component (424), and Molecular Function (1449) (Table S5). Among the annotated DEGs, the most significantly enriched GO terms included: transcription regulation, signal transduction, cell wall organization, trans-membrane transport, an integral component of membrane, nucleus, cytoplasm, ATP binding, metal ion binding, DNA binding activities, various kinase activities, and trans-membrane transporter activities (Figure S5). In addition, the functional annotation of unigenes that was based on GO categorization of lentil and *Rhizoctonia* transcriptome revealed many significantly enriched GO terms viz., membrane, membrane part, antioxidant activity, transcription regulation, response to biotic stimulus, etc. (Figure 2).

**Figure 3:**
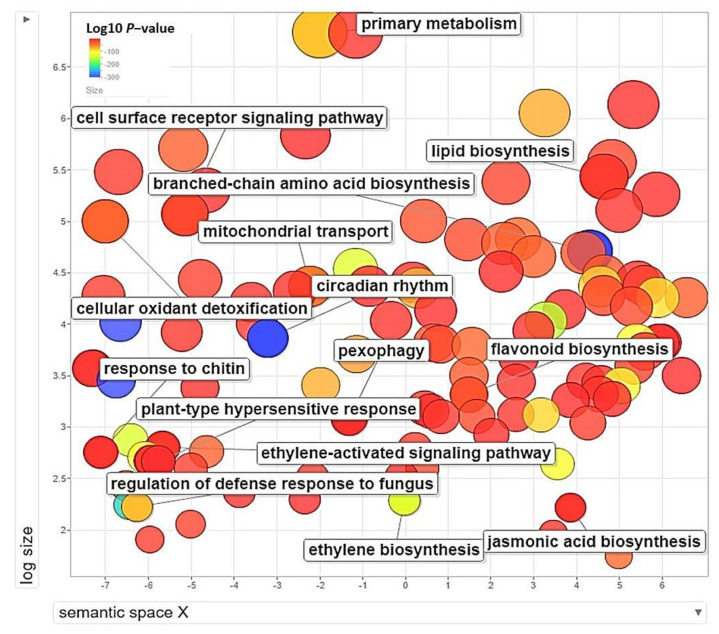
Scatterplot showing the over-represented GO term (*p* < 0.01) in all comparisons with labels on the disease responsive terms. Different shades in circles indicate the difference in *p*-values (as given in the scale) whereas the bubble size indicates the frequency of the GO term.

All the DEGs with GO allotted (3169) across all the samples were subjected to gene enrichment analysis and 93 GO terms got enriched. The enriched GO terms are further visualized as a scatterplot (Figure 3). Among the various GO terms, ‘response to Chitin’ (GO:0010200), plant HR (GO:0009626), cell surface receptor signaling pathway (GO:0007166), and ‘JA biosynthesis’ (GO:0009695) are significantly enriched which supported the hypothesis of operation of a JA/ET-mediated response pathway during lentil-fungus interaction.

**Figure 4:**
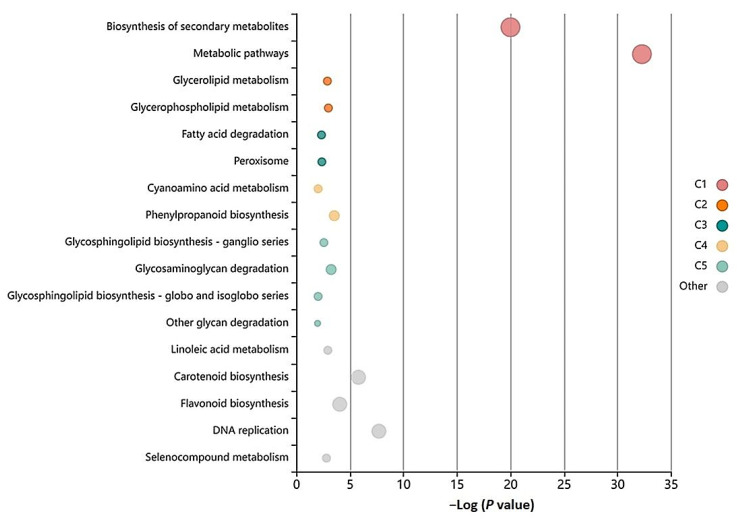
Bubble plot showing KEGG enriched terms for lentil genotype Precoz when infected with *Rhizoctonia* where each bubble represents an enriched function and the size of the bubble from small to large represents their relative abundance. The color of the bubble is the same as the color in the network, which represents a different cluster. For each cluster, if there are more than five terms, the top five with highest enrich ratio are displayed.

To identify the major biological pathways which got altered in *L.* *culinaris* when infected with *Rhizoctonia*, the core DEGs were mapped to the KEGG pathways database. The KEGG annotated unigenes (10,604) were distributed to 109 KEGG pathways (Table S6); among these pathways, the over-represented pathways include ‘metabolic pathways’, ‘secondary metabolite biosynthesis’, ‘DNA replication’, ‘glycerolipid metabolism’, ‘fatty acid degradation’, and ‘carotenoid and flavonoid biosynthesis’ pathways (Figure 4)

### 3.6. PHI-Base: Pathogen Host Interaction 

PHI-base analysis was performed to find the molecular and biological information on the key genes affecting *Rhizoctonia*-lentil interactions including the information on the target sites of some defense anti-infective chemistry. The PHI-base hits identified a total of 725 transcripts as gene homologues that are involved in pathogen-host interactions which included both compatible and incompatible reactions (Table S7). These genes are further classified into five major categories viz. reduced virulence (45.66%), unaffected pathogenicity (28.69%), loss of pathogenicity (12.69%), lethal (6.48%), increased virulence (3.17%), effector (2.34%), resistance to chemical (0.55%), and sensitivity to chemical (0.41%) (Table 3).

### 3.7. miRNA Prediction, miRNA Target Site Identification, and Functional Analysis

Through homology searches of the sequences and miRNA prediction with the c-mii program, we could identify a total of 354 unique miRNA candidates. The putative miRNAs varied from 18–24 nucleotides in length with a majority of them being 21 nt in length. The MIR169 family was the largest family with 71 members followed by mir156, mir159, mir167, mir319, mir160, and mir164 (Tables S8 and S9). Among these a few stress-related miRNAs viz., miR156, miR159, miR167, miR169, miR482, etc. were found to have biotic stress-related functions such as the formation of disease resistance proteins. This study, for the first time, could identify the transcriptome-based miRNAs and their targets in response to *Rhizoctonia* infection in lentils which can be used for further analysis of miRNAs and their role in *Rhizoctonia* stress response in lentils. 

### 3.8. PPI Network Analysis 

**Figure 5:**
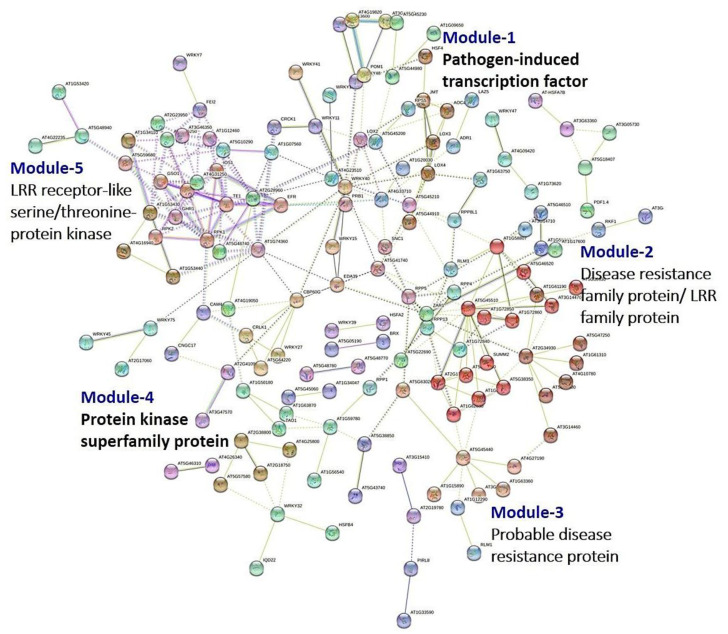
PPI network of lentil genotype Precoz when infected with *R. bataticola*.

The STRING analysis was used to find the tightly connected regions in the PPI network representing molecular complexes in susceptible dry root rot lentil genotype Precoz under infected and control conditions. The predicted PPI network was broadly divided into five major functional modules which corresponded to respective pathways (Figure 5). Module-1 consisted of proteins that were involved in pathogen-induced TF activity, while Module-2 consisted of disease resistance family proteins/LRR family proteins. Module-3 consisted of probable disease resistance proteins and Module-4contained mainly protein kinase superfamily proteins. Module-5contained LRR receptor-like serine/threonine-protein kinase. It was interesting to note that even under susceptible expression of Precoz (when infected with *Rhizoctonia*), the resistance imparting pathways such as kinase and other resistance family proteins (LRR family) got induced. 

### 3.9. Validation Studies Using InSilico Expression Analysis

**Figure 6:**
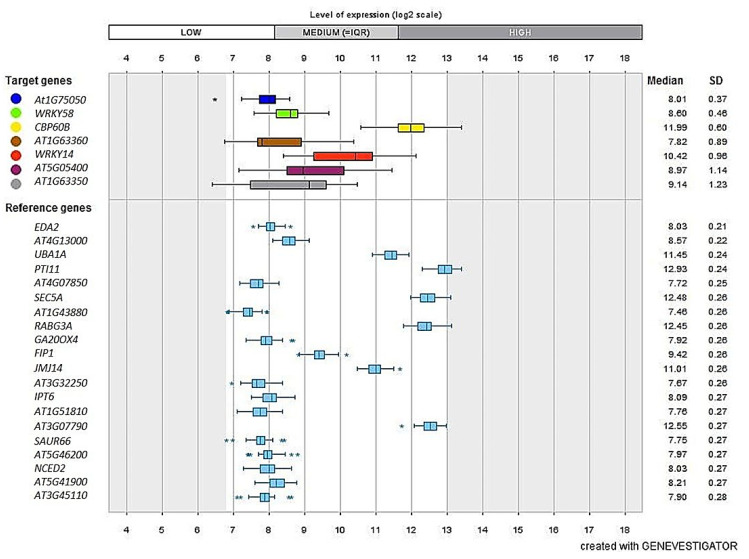
A total of 20 *Arabidopsis* reference genes showed correspondence in the expression levels with that of seven selected target genes as obtained after infection of lentil with *Rhizoctonia*. * and ** shows that the expression is significant (*p* ≤ 0.05) and highly significant (*p* ≤ 0.01), respectively.

Genevestigator is a high-quality database having tools to generate novel information about gene expression such as when, where, and how the genes get expressed in a living system [47]. This helps in the validation of the obtained results and also generation of a new hypothesis. Initially, seven genes could be identified from the lentil-*Rhizoctonia* RNA-seq data which got validated against *A.* *thaliana* under the infected vs. control comparison (Figure 6). These seven target genes viz., AT1G75050 (PR superfamily protein), WRKY58, WRKY14, (WRKY DNA-binding protein), CPB60B, AT5G05400 (disease resistance), AT1G63360, and AT1G63350 (NBS-LRR class disease resistance protein) got validated with those of 20 Arabidopsis reference genes viz., AT4G13000, PTI11, AT5G46200, AT1G51810 (kinase family protein), GA20OX4, SAUR66, NCED2, IPT6, AT3G32250 (hormone-related proteins), EDA2 (Probable serine protease), UBA1A (UBP1-associated proteins 1A), SEC5A (Exocyst complex component SEC5A), FIP1 (root response to stress), JMJ14 (transcriptional gene regulation), AT3G07790 (DGCR14-like protein), AT1G43880, AT3G45110, AT4G07850 (hypothetical protein), RABG3A (Signal transduction), and AT5G41900 (hydrolase activity) (Figure 6).

**Figure 7:**
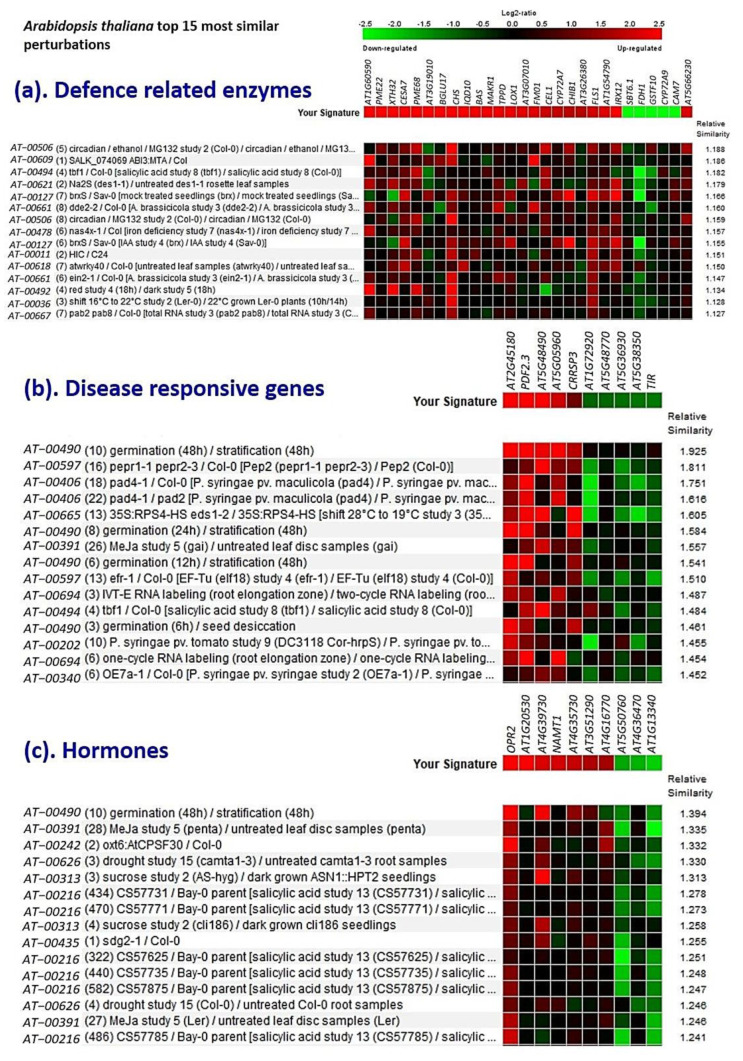

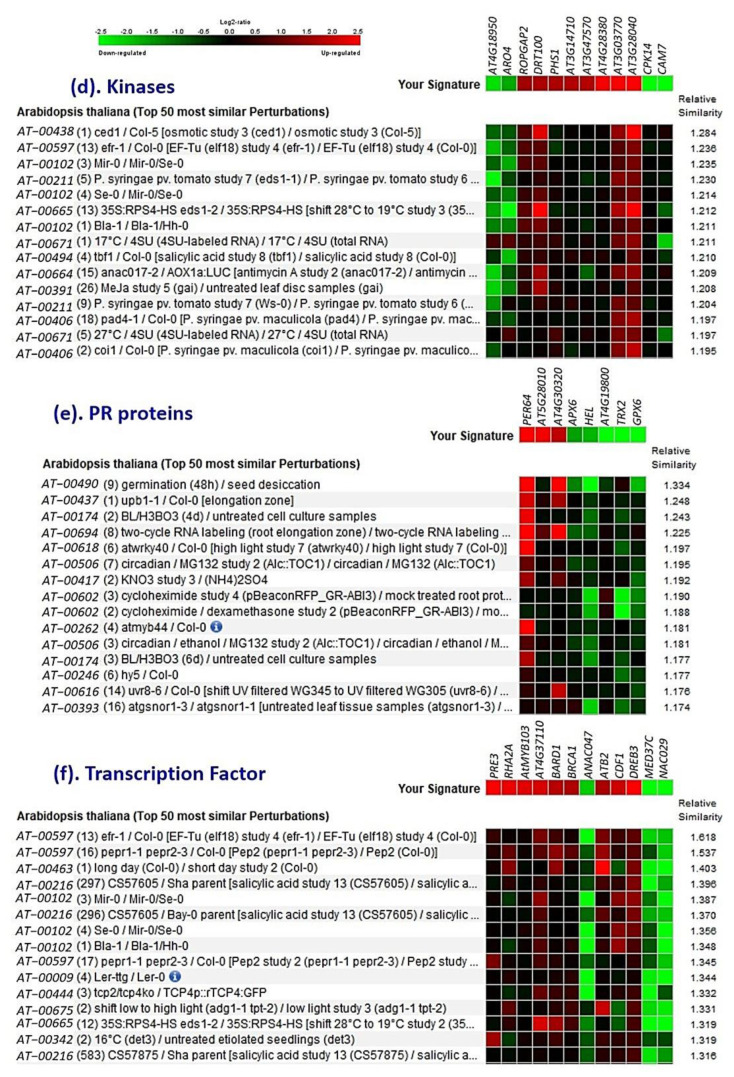
Heat-map that was generated using the DEGs that were associated with (**a**) defense-related enzymes, (**b**) disease responsive genes, (**c**) hormones, (**d**) kinases, (**e**) PR proteins, and (**f**) Transcription factors during lentil-*Rhizoctonia* interaction. Genevestigator was used and the top 15 perturbations are presented.

In addition, several genes that were identified from the lentil-*Rhizoctonia* interaction and belonging to six major defense response groups viz., defense-related enzymes, disease responsive genes, hormones, kinases, PR proteins, and TF were also validated with that of *A. thaliana* and the top 15 major perturbations are presented in Figure 7 as a heat map. Interestingly, for the defense-related enzymes, hormones, and TF category of DEGs, salicylic acid (SA) was found to be correlated. Whereas for disease-responsive genes, kinases, and PR proteins categories of DEGs, germination-related pathway genes could be mainly validated (Figure 7).

## 4. Discussion

### 4.1. Gene Expression and Validation 

Since lentil sequence information is still incomplete with minimum annotation and limited access [25], we, therefore, opted for denovo analysis. In this study, we have generated a total of 452 million reads which is considered enough to do the detailed analysis. Similarly, Hosseini et al. [50] also generated 404.67 million reads while studying the abiotic stress tolerance in lentil. RNA-Seq data showed the differential expression of several defense-related genes with high fold change values and were classified into six main groups viz., defense-related enzymes, disease responsive genes, hormones, kinases, PR (pathogenesis related) proteins, and TF. Similarly, the key metabolic pathways that were identified in peanut for stem rot infection also include ‘DNA replication’, ‘photosynthesis’, ‘carbon metabolism’, etc. [23]. For PHI-base analysis, Dasgupta et al. [24] identified many transcripts as gene homologues which were found to be associated with both compatible and incompatible host-pathogen interactions (reduced virulence, unaffected pathogenicity, loss of pathogenicity, etc.) which is similar to what we have recorded in lentil when infected with *Rhizoctonia*. 

**Figure 8:**
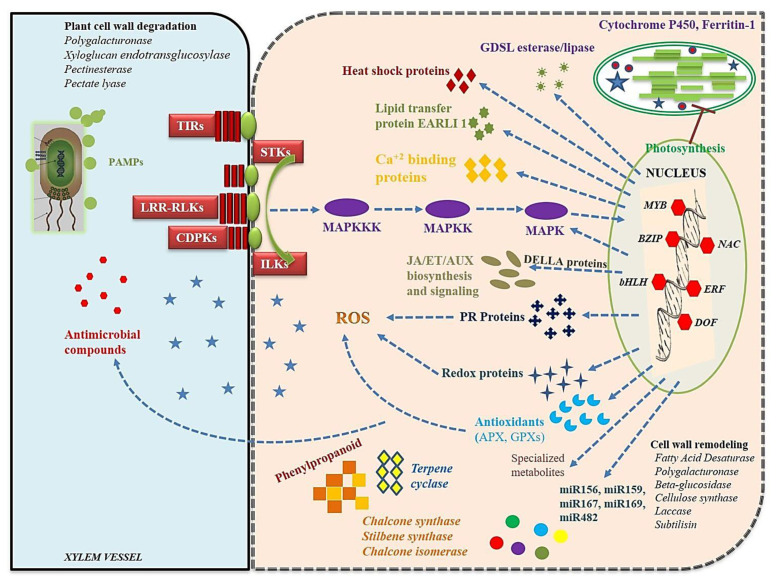
The molecular network operating in *L.* *culinaris* when infected with *R.* *bataticola*. Comparative transcriptomic analysis has shown the perception of fungus by lentil root cells resulting in the degradation of the cell wall and its establishment by taking control over the host defense. Where, APX: Ascorbate peroxidase; AUX: auxins; CDPK: calmodulin-dependent protein kinase; ET: ethylene; ERF: ethylene response factor; GPX: Glutathione peroxidase; GDSL: consensus amino acid sequence of Gly, Asp, Ser, and Leu around the active site Ser; ILKs: Integrin-linked kinases; JA: jasmonic acid; LRR-RLKs: leucine-rich repeat receptor-like kinases; MAPKs: mitogen-activated protein kinases; PAMPs: pathogen-associated molecular patterns; PR proteins: pathogenesis-related proteins; ROS: Reactive oxygen species; STKs: receptor-like serine/threonine-protein kinases; Transcription factors: WRKY, MYB, NAC, bHLH, etc.

As identified in this study, the PPI network analysis in mungbean also identified several modules consisting of proteins that are involved in kinase, hydrolase, DNA binding, etc. activities when infected with MYMIV [24]. Insilico validation analysis was performed on the DEGs (infected vs. control) using *A.* *thaliana* as a reference through Genevestigator^®^ [47] and many defense-related enzymes, kinases, hormones, and TFs were identified as also reported by Dam et al. [51] while studying the gene co-expression analysis for disease gene prediction. To get a meaningful conclusion, we have systematically analyzed the RNA-Seq data starting from pathogen recognition to disease expression which is thoroughly discussed in the following sections. Also, an intricate molecular network that was found operating in *L. culinaris* when infected with *R. bataticola* is formed and presented as Figure 8.

### 4.2. Recognition of Rhizoctonia and Expression of Plant Signaling Pathways

After the infection of lentil by *Rhizoctonia*, many protein kinases such as LRR receptor kinase (LRR-RK) and calmodulin-dependent protein kinase (CDPK) were identified playing a key role in recognition and early signaling. The LRR in LRR-RK and serine/threonine kinase-like domain in CDK are reportedly associated with pathogen recognition and signaling, respectively, during host-pathogen interactions [31,52,53]. Additionally, DEGs that are associated with plant hormones pathways such as ET-responsive transcription factor (ERF), JA, and SA signaling pathways seem to contribute a major role in the regulation of host-pathogen response mechanisms against the fungal infection [54]. In this study too, several DEGs encoding SA, ET, JA, and auxin (AUX) signaling pathways such as SA carboxyl methyltransferase, ERF, allene oxide cyclase (associated with JA biosynthesis), AUX-induced protein, AUX conjugate hydrolase, etc. were identified which are involved in induced systemic defense responses in plants. The activation of ERF is known to positively regulate the expression of Calcium/CDPK, and is also reported in *Sclerotinia sclerotiorum*-infected tomatoes [55] and in *A. lentis*-infected lentil [31]. Since the CDPK showed a very similar expression pattern to that of ERF, thus, the CDPK-like DEGs also appears as an important signaling molecule once *Rhizoctonia* invades the lentil roots. LRR-RK showed a comparable expression pattern with that of ERF and CDPK in the infected lentil plant and activates the ERF by triggering the CDPK-like genes [52,53].

Most of the genes that are involved in ET/JA biosynthesis were found to be up-regulated while a majority of the genes that are involved in SA/AUX biosynthesis were found down-regulated in the infected plants when compared with the mock-inoculated or control plants (Table S1). Further, in the process of host-pathogen interaction, the DEGs of chalcone synthase, chalcone/stilbene synthase, chalcone-flavanone isomerase, and terpene cyclase/mutase were found to be up-regulated with high fold change and are involved in phenylpropanoid biosynthesis that triggers defense responses in lentil [23]. In plants, receptor-like kinases (RLKs), which are a large superfamily of PRRs proteins, are involved in various plant responses including a plants’ response against pathogen infection [30,56]. This study could also identify various differentially regulated protein kinases such as LRR-RLKs, toll/interleukin-1 receptor-like protein (TIR), integrin-linked kinase family protein (ILK), serine/threonine kinase catalytic domain protein (STK), calmodulin-binding protein, and CDPKs that are known to be associated with the PRRs that activate the mitogen-activated protein kinase (MAPK) signaling cascade/networks [23,24] which results in various defense mechanisms in lentil plants against *Rhizoctonia* infection. 

### 4.3. Structural and Biochemical Response to Rhizoctonia Infection

Besides signaling, other structural and biochemical responses were also identified after infection. At the point of fungal penetration in the lentil, a physical barrier or papilla is formed [31,57]. In lentils, the accumulation of xyloglucan endotransglucosylase/hydrolase (XTH) is known in response to the *A. lentis* infection, which functions in elongation and restructuring of the cell wall [31]. Elevated XTH levels in our study also suggest the response to *Rhizoctonia* infection and preparation for the physical barrier formation. In addition, laccase (a structural gene) was also found to be upregulated under *Rhizoctonia*-infected conditions as also observed in lentil when infected by *Ascochyta* [57]. This helps in the unrestricted movement of the pathogen via poor or no cross-linking of cell wall polymers [58]. Fatty acid desaturase (FAD) is another key structural gene that was found to be differentially expressed under infection. FADis mainly associated with the cell function maintenance by regulation of tissue fatty acid profile maintenance under various stresses including *A. lentis* infection in lentil [31]. In addition, several other DEGs encoding cell-wall degrading enzymes viz., polygalacturonase, pectinesterase, β-glucosidase, pectate lyase, etc. were observed which seem to be responsible for the degradation of lentil root cell wall during fungal infection.

Among various biochemical defense responses, PR proteins and ROS (reactive oxygen species) are the most common anti-fungal compounds that were identified in lentils when infected with *Ascochyta* [59,60]. Genes that were related with other antioxidant properties (e.g., peroxidase, glutathione peroxidase, L-ascorbate peroxidase, and glutathione-S-transferase) were also identified to be highly up-regulated (Table S3). The most prominent up-regulated PR genes includedPR1, PR2, PR5, PR10, and PR bet V I in response to *Rhizoctonia* infection. Similarly, PR2 proteins are known to cause fungal cell wall hydrolysis in *Fusarium oxysporum*-infected chickpea [61]. In addition, these proteins are also involved in the release of various elicitors from the fungal cell wall which is then recognized by the receptors that are present on the lentil roots which cause the activation of various downstream signaling responses [62]. PR4 was reportedly involved in the disruption of the fungal cell wall polarity and fungal growth regulation in lentil [63]. PR10 acts by expressing the RNase activity on the invading fungal hyphae [64]. Thus, all these seem to play a very important role during host-pathogen interactions in a complex and integrated manner.

Several other DEGs were identified which code for various enzymes having antifungal activities such as plant invertase/pectin methylesterase inhibitor (PMEI), polygalacturonase inhibitor-like protein (PGIP), and AUX-repressed 12.5 kDa protein (ARP). PMEI functions by reducing the susceptibility of the plant cell wall to fungal endopolygalacturonases [65], while PGIP functions by specific binding which restricts the damage potential of fungal polygalacturonases [66]. ARP functions by inhibiting the pathogen through AUX production manipulation [67]. Many genes causing the initiation of the defense response under infection were also significantly induced. The genes such as Cytochrome P450 4A25 (involved in the oxidation of plant hormone jasmonoyl-L-isoleucine), heat shock protein HSS1, andlipid transfer protein EARLI 1-like were found to be up-regulated under infected conditions as also reported for stem rot (*Sclerotium rolfsii*) infection in peanut [23]. 

### 4.4. Hypersensitive Response (HR), Cell Death, and Systemic Acquired Resistance (SAR)

The NB-ARC domain protein (of NB-LRRs) regulates the signal transduction resulting in HR signaling and finally cell death [68]. Besides, E3 ubiquitin-protein ligase, which has its role in HR, also got overexpressed in lentils as also reported for potatoes when infected with *Phytophthora* [69]. SAR is induced by either some signal molecule or hormone and gives long-lasting protection against the pathogen along with an HR [70]. A few putative SAR-associated genes, viz., putative gibberellin signaling DELLA protein LA, Gibberellin-regulated family protein, and SCF ubiquitin ligase showed upregulation upon *Rhizoctonia* infection. The DELLA proteins are known to impart defense to fungal infection via JA/ethylene-dependent defense response activation along with the regulation of overall ROS contents in the plant under infection [71].

### 4.5. TFs Related to Fungal RESPONSES

Interestingly, the present study identified several differentially regulated TFs families and the most prominent ones are AP2 domain, bHLH, DOF, MYB, NAC, ERF, BZIP, etc. which act as regulators in plant defense mechanisms during *Rhizoctonia* infection. The differential regulation of such TFs are also reported for various biotic and abiotic stress in plants by Vidhyasekaran [72], and also for *S.* *rolfsii* and MYMIV infection in peanut [23] and mungbean [24], respectively. A large number of mentioned TF were downregulated and only a few of these categories were upregulated when checked under infection. The downregulation of ERF was recorded which is involved in the stimulation of PR protein expression [73], indicating a lack of defense in the susceptible genotype Precoz to *Rhizoctonia* infection. Also, the data identified several differentially expressed kinases (viz, RLK), PR proteins, WRKY TFs, etc. as also reported in rice when infected with *Ustilaginoidea virens* [74].

### 4.6. Role of miRNAs

Several diverse biological processes including biotic stress tolerance have been known to be regulated by an array of miRNAs via the regulation of certain gene expression at the post-transcriptional level. Therefore, the identification of miRNA target sites from any RNA-seq data is of utmost importance to understand the probable roles of these putative miRNAs in the post-transcriptional regulation of some key DEGs [75]. Recently, deep learning (DL) was found as a very effective way as it can autonomously learn and identify the miRNA target sites from the raw RNA-Seq data [76]. The identification of miRNA targets from the RNA-Seq data were mostly explored for the animal systems [77]; however, in *Typha angustifolia* under Cd stress, a total of 764 miRNA target sites could be identified of which 21 were found as novel miRNAs [78]. Similarly, in our transcriptomic studies too, we have identified the differential expression of some miRNA target sites, of which miR156, miR159, miR167, miR169, and miR482 were found as the major ones having their role in the disease response in the lentil. Interestingly, in soybean, many miRNAs have been found to have a role in the biotic stress tolerance, and resistance to soybean mosaic virus (SMV) infection was reportedly being regulated by several miRNAs such asmiR156, miR159, miR167, and miR169 [79]. In addition, miR156 was also found to have a role in the imposition of resistance against bean pyralid larvae infestation [80]. The miR169 showed its role when soybean gets infected with rust (*Phakopsorapachyrhizi*), soybean cyst nematode [81], and MYMIV [82]. Similarly, miR482 showed differential expression in soybean when infected with rust [83]. In the future, detailed studies are required to establish the exact role of these miRNAs in the dry root disease response in the lentil. 

## 5. Conclusions

The RNA-Seq analysis has conclusively shown that the *R.* *bataticola* infection in lentils triggers the expression of a complex gene network which helps in the establishment of fungal growth in the roots of lentil plant. This intricate gene network begins with the formation of PAMPs which is followed by the activation of different kinase-signaling pathways which result in the formation of various proteins that then result in the establishment of *Rhizoctonia* infection in the lentil roots (Figure 8). Various signaling cascades, along with the differential expression of different TFs, caused the formation of many defense-related PR-proteins, antioxidative enzymes, cell-wall degrading and remodeling enzymes, miRNAs, and other secondary metabolites. Such information will be of great help in the identification of various key host genes that are associated with the expression of disease symptoms and help in a detailed understanding of lentil-*Rhizoctonia* interactions resulting in a compatible reaction. This study has unfolded an intricate and coordinated expression of a complex gene network, which has not only resulted in an insight into the disease expression mechanism but also revealed a novel understanding of the gene networks resulting in dry root rot infection in lentils. Thus, the information that was generated about the key genes and pathways causing compatible interactions (of *Rhizoctonia* with lentils) can be used to modify them for the development of lentil genotypes that are resistant to dry root rot.

## Figures and Tables

**Table 1:** Summary of RNA-seq data in four lentil root samples (control and infected).

Parameters	Sample Name			
	Inoculated (Root-A1)	Inoculated (Root-A2)	Control (Root-B1)	Control (Root-B2)
Read length	100 × 2	100 × 2	100 × 2	100 × 2
Raw reads	110,534,542	107,467,648	119,708,340	114,381,960
Clean read	110,235,896	107,170,090	119,341,370	113,806,616
Clean bases (Mb)	10,981.28	10,662.28	11,879.05	11,343.24
GC contents (%)	43.07	42.88	44.01	43.95
Q30 (%)	93.64	94.23	93.84	94.12
Total No. of paired end (PE) reads	55,117,948	53,585,045	59,670,685	56,903,308
Read alignment (%)	96.82	97.06	96.93	97.05
Unigenes (No) with FPKM ≥ 1	37,242	37,631	36,421	37,419

Note: Where A1and A2: Infected (fungal inoculated), B1 and B2: Control.

**Table 2:** BLASTX, UniProt, and Unigene Annotation summary.

Description/Annotation Category	Number of Unigenes
Total number of Unigenes considered for BLASTX	42,606
Number of Unigenes with significant BLASTX match	38,437
Number of Unigenes with a significant BLASTX hit against (*Rhizoctonia* protein sequences)	12,648
Number of Unigenes with a significant BLASTX hit against (Uniprot plant protein database)	25,789
Number of unigenes with No Blastx hit	4169

**Table 3:** PHIBase analysis of DEGs identified in response to fungus infection in lentil.

Categories	Count	Percentage
Reduced virulence	331	45.66
Unaffected pathogenicity	208	28.69
Loss of pathogenicity	92	12.69
lethal	47	6.48
Increased virulence (hypervirulence)	23	3.17
Effector (plant avirulence determinant)	17	2.34
Resistance to chemical	4	0.55
Sensitivity to chemical	3	0.41
Total	725	100

## Data Availability

Data sharing is not applicable.

## Supplementary Materials

The following are available online at https://www.mdpi.com/article/10.3390/genes13010090/s1, Figure S1: (A) and (B) Hypha of *R. bataticola* (Accession No. OL304938) stained with lactophenol showing typical right-angled branching and constriction at the base of the hyphal branches at 10× and 20×magnification respectively; (C) and (D) Sclerotia of the fungus at 10× magnification under compound microscope, Figure S2: (a) The length distribution details of the assembled transcript and unigene; (b) Transcript expression (FPKM) distribution of all the samples; (c) Transcript expression that was based on the PCA analysis showing correlation between the sample replications. Figure S3: (a) The total number of the upregulated and downregulated DEGs (with *p* ≤ 0.05 & Log2 FC +2/−2) that were identified in the lentil genotype Precoz under the control and infected conditions; (b) MA plot, (c) Volcano plot, and (d) FPKM plot of the DEGs that were identified in the lentil genotype Precoz under control and infected conditions. Figure S4: BLASTX hit of top 25 organisms. Figure S5: Some of the selected GO terms in (a) biological process, (b) cellular component, and (c) molecular function category from GO annotation of lentil transcriptome. Table S1: The assembled transcript summary. Table S2: The transcript expression and total number of transcripts (length ≥200 bp). Table S3: Annotation details using Uniprot. Table S4: Annotations details of the total DEGs with that of *Rhizoctonia*. Table S5: The categorization of GO terms into three major GO classes (MF, BP and CC). Table S6: KEGG pathway analysis. Table S7: PHI-base analysis of the lentil RNA Seq data. Table S8: List of miRNA target sites identified from the RNA Seq data. Table S9: Stress-associated miRNA target site details.
