# Supplementary material for: Insights into the Host-Pathogen Interaction Pathways through RNA-Seq Analysis of Lens culinaris Medik. in Response to Rhizoctonia bataticola Infection
Source: Genes (Basel). 2021 Dec 29;13(1):90. doi: 10.3390/genes13010090 (PMC8774501; doi:10.3390/genes13010090)
Supplement: Supplementary file 1 [file genes-13-00090-s001.zip › Supplementary File Figures.pdf]

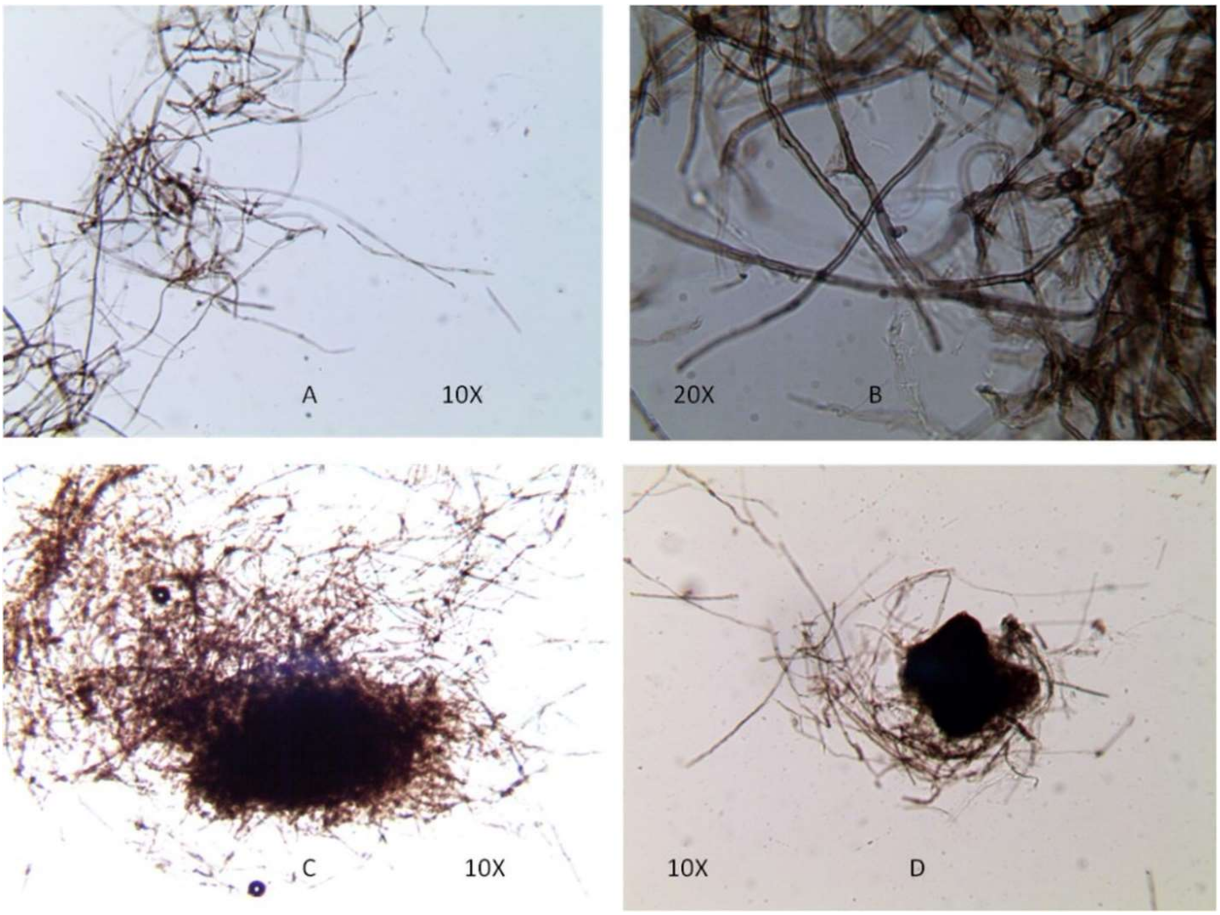

Figure S1. **(A) and (B) Hypha of *R. bataticola* (Accession No. OL304938) stained with lactophenol showing typical right-angled branching and constriction at the base of the hyphal branches at 10X and 20X magnification respectively; (C) and (D) Sclerotia of the fungus at 10X magnification under compound microscope.**

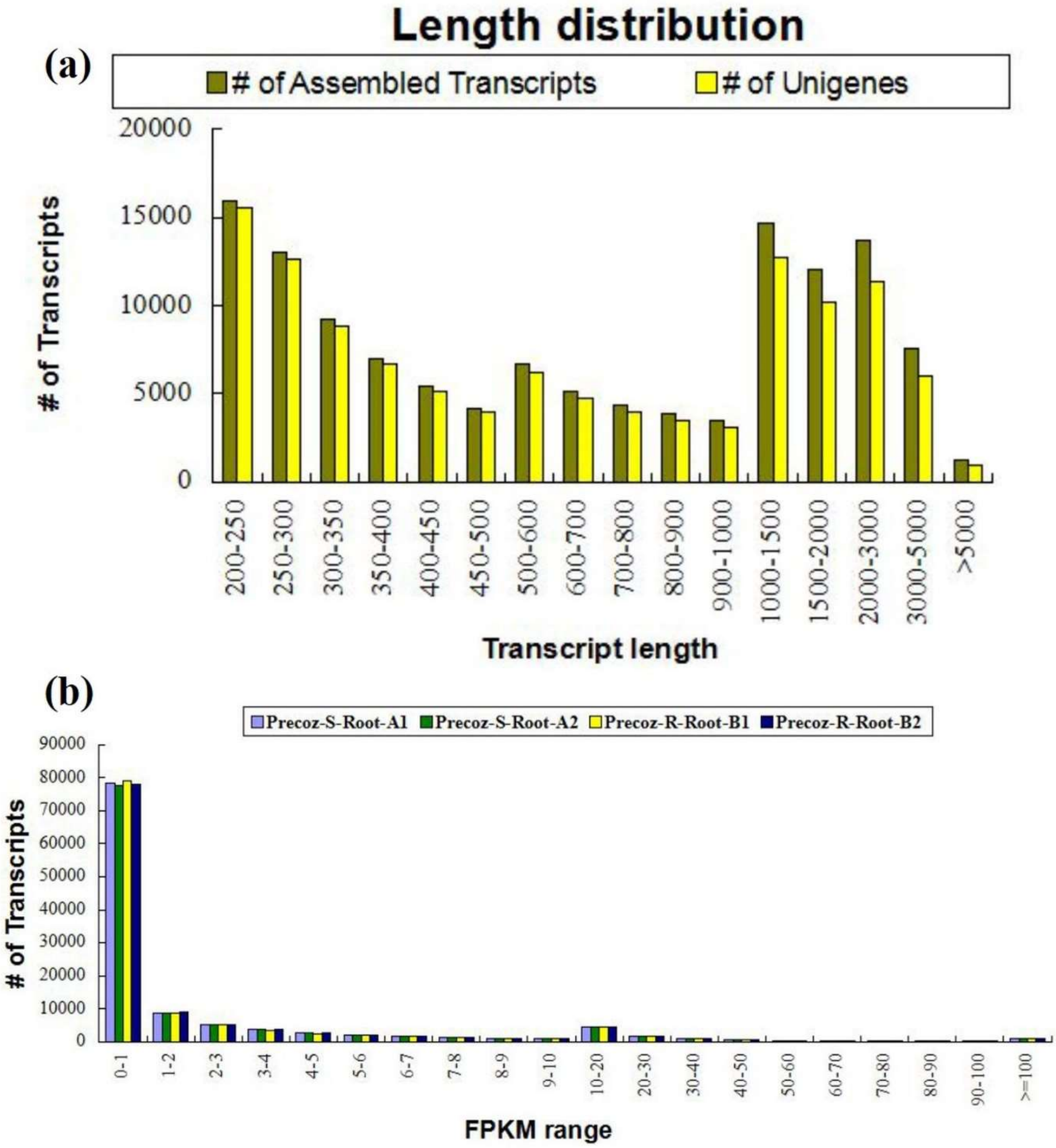

Figure S2. (a) The length distribution details of the assembled transcript and unigene; (b) Transcript expression (FPKM) distribution of all the samples.

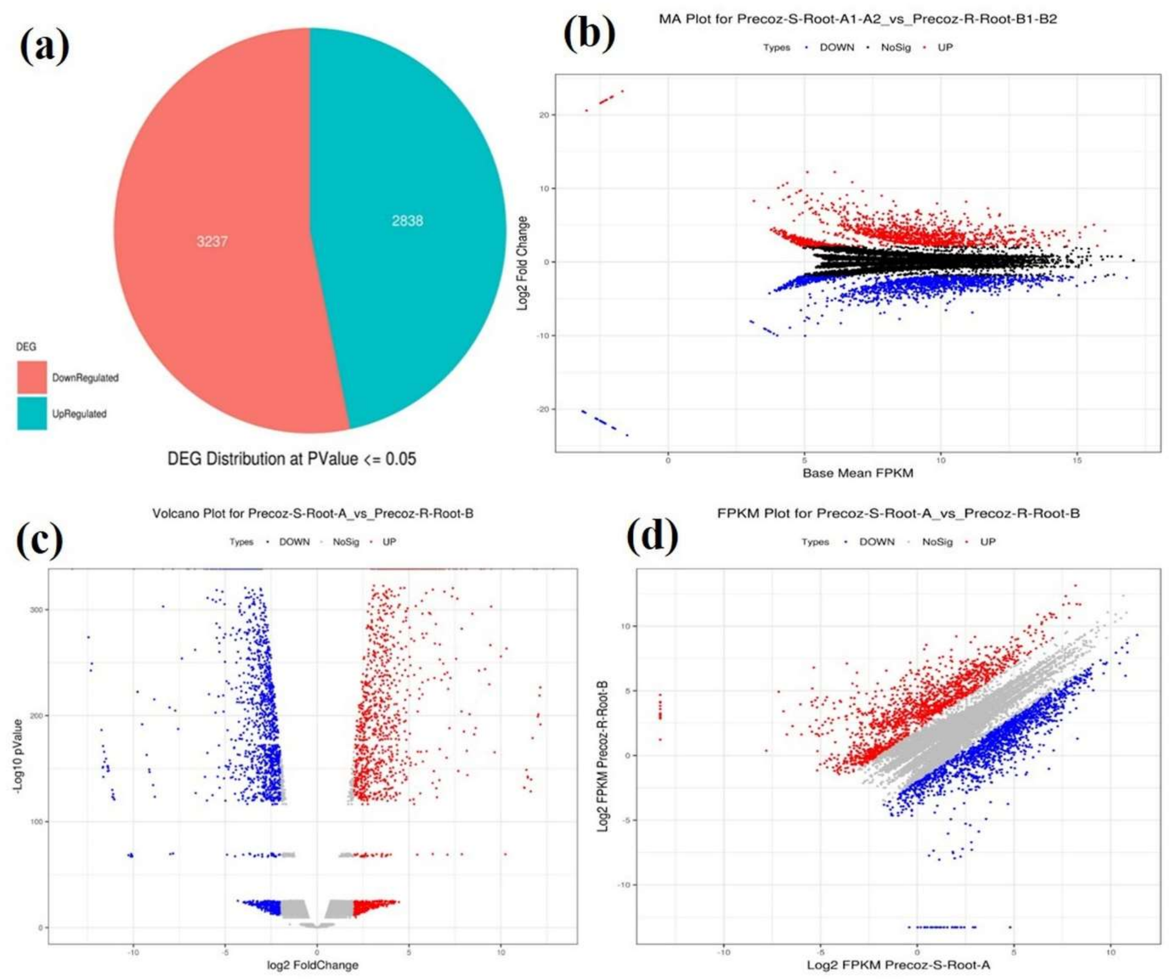

Figure S3. (a) Total number of upregulated and downregulated DEGs (with p≤0.05 & Log2 FC +2/-2) identified in the lentil genotype Precoz under control and infected conditions; (b) MA plot, (c) Volcano plot and (d) FPKM plot of the DEGs identified in the lentil genotype Precoz under control and infected conditions.

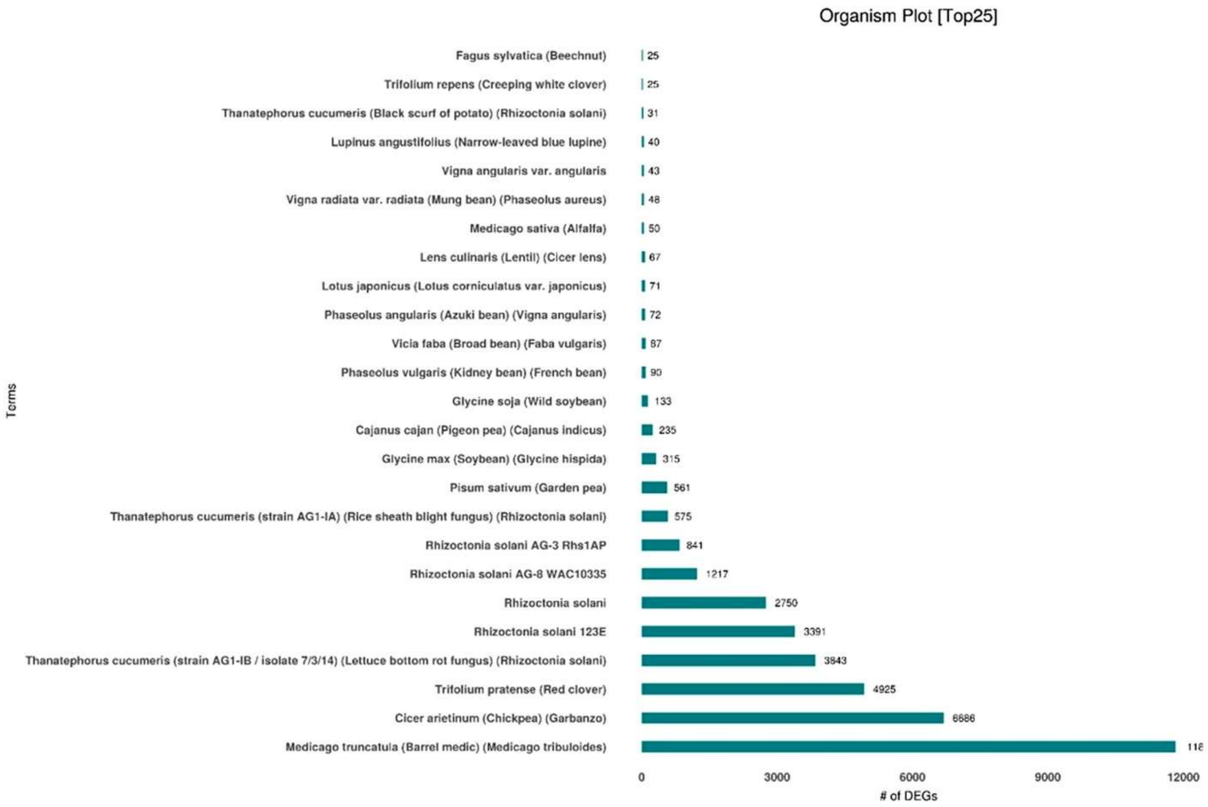

Figure S4. BLASTX hit of top 25 organisms.

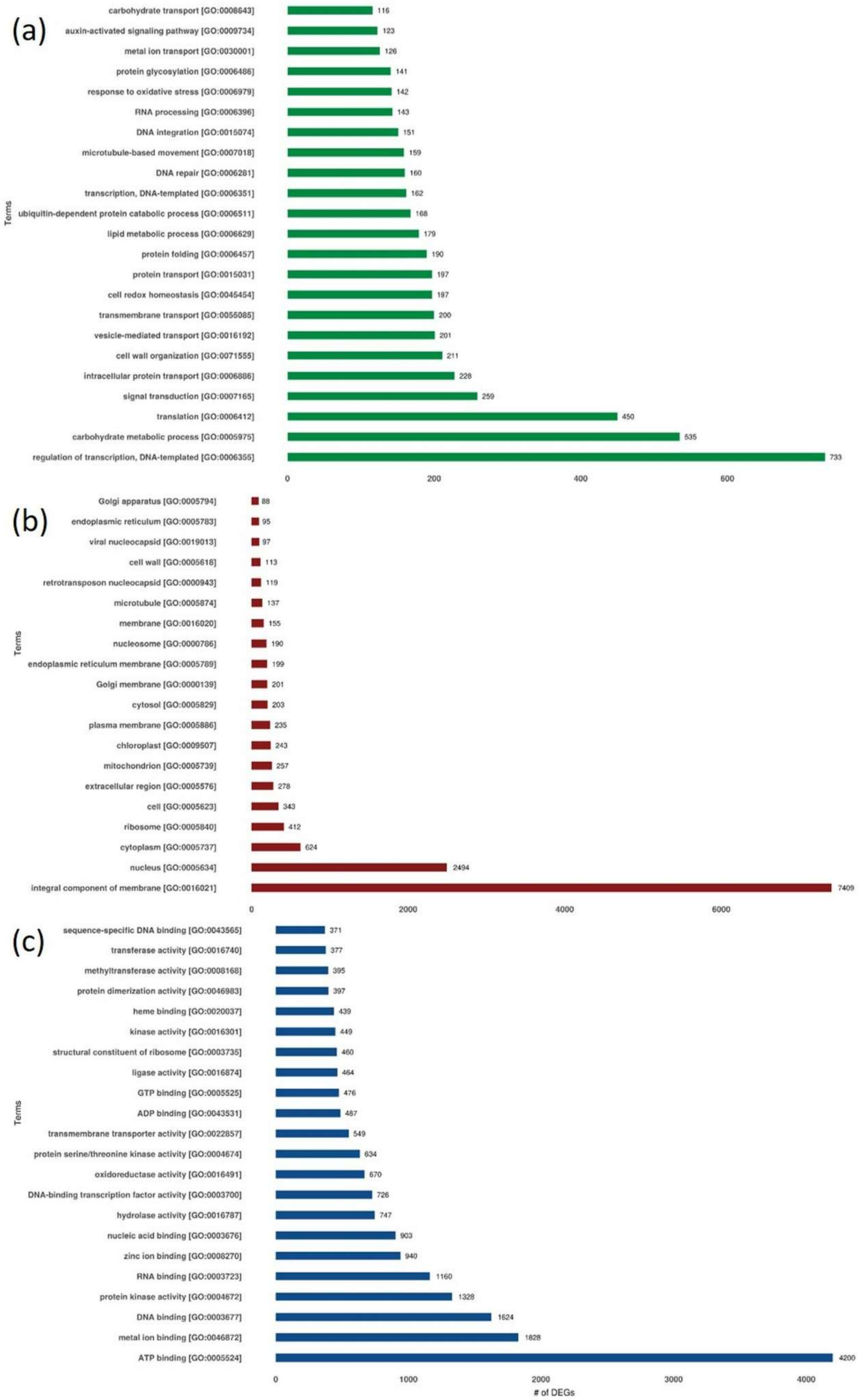

(a)
carbohydrate transport [GO:0008643] 116
auxin-activated signaling pathway [GO:0009734] 123
metal ion transport [GO:0030001] 126
protein glycosylation [GO:0006486] 141
response to oxidative stress [GO:0006979] 142
RNA processing [GO:0006396] 143
DNA integration [GO:0015074] 151
microtubule-based movement [GO:0007018] 159
DNA repair [GO:0006281] 160
transcription, DNA-templated [GO:0006351] 162
ubiquitin-dependent protein catabolic process [GO:0006511] 168
lipid metabolic process [GO:0006629] 179
protein folding [GO:0006457] 190
protein transport [GO:0015031] 197
cell redox homeostasis [GO:0045454] 197
transmembrane transport [GO:0055085] 200
vesicle-mediated transport [GO:0016192] 201
cell wall organization [GO:0071555] 211
intracellular protein transport [GO:0006886] 228
signal transduction [GO:0007165] 259
translation [GO:0006412] 450
carbohydrate metabolic process [GO:0005975] 535
regulation of transcription, DNA-templated [GO:0006355] 733
Terms
0 200 400 600
(b)
Golgi apparatus [GO:0005794] 88
endoplasmic reticulum [GO:0005783] 95
viral nucleocapsid [GO:0019013] 97
cell wall [GO:0005618] 113
retrotransposon nucleocapsid [GO:0000943] 119
microtubule [GO:0005874] 137
membrane [GO:0016020] 155
nucleosome [GO:0000786] 190
endoplasmic reticulum membrane [GO:0005789] 199
Golgi membrane [GO:0000139] 201
cytosol [GO:0005829] 203
plasma membrane [GO:0005886] 235
chloroplast [GO:0009507] 243
mitochondrion [GO:0005739] 257
extracellular region [GO:0005576] 278
cell [GO:0005623] 343
ribosome [GO:0005840] 412
cytoplasm [GO:0005737] 624
nucleus [GO:0005634] 2494
integral component of membrane [GO:0016021] 7409
Terms
0 2000 4000 6000
(c)
sequence-specific DNA binding [GO:0043565] 371
transferase activity [GO:0016740] 377
methyltransferase activity [GO:0008168] 395
protein dimerization activity [GO:0046983] 397
heme binding [GO:0020037] 439
kinase activity [GO:0016301] 449
structural constituent of ribosome [GO:0003735] 460
ligase activity [GO:0016874] 464
GTP binding [GO:0005525] 476
ADP binding [GO:0043531] 487
transmembrane transporter activity [GO:0022857] 549
protein serine/threonine kinase activity [GO:0004674] 634
oxidoreductase activity [GO:0016491] 670
DNA-binding transcription factor activity [GO:0003700] 726
hydrolase activity [GO:0016787] 747
nucleic acid binding [GO:0003676] 903
zinc ion binding [GO:0008270] 940
RNA binding [GO:0003723] 1160
protein kinase activity [GO:0004672] 1328
DNA binding [GO:0003677] 1624
metal ion binding [GO:0046872] 1828
ATP binding [GO:0005524] 4200
Terms
0 1000 2000 3000 4000
# of DEGs

Figure S5. Some of the selected GO terms in (a) Biological process, (b) Cellular component, (c) Molecular function category from GO annotation of lentil transcriptome.
