# Supplementary material for: Insights into the Host-Pathogen Interaction Pathways through RNA-Seq Analysis of Lens culinaris Medik. in Response to Rhizoctonia bataticola Infection
Source: Genes (Basel). 2021 Dec 29;13(1):90. doi: 10.3390/genes13010090 (PMC8774501; doi:10.3390/genes13010090)
Supplement: Supplementary file 1 [file genes-13-00090-s001.zip › Table S2. Transcript expression and total number of transcripts (length í▌200bp)..pdf]

Table S2. Transcript expression and total number of transcripts (length ≥200bp).

FPKM Range	Samples			
	Precoz-S-Root-A1	Precoz-S-Root-A2	Precoz-R-Root-B1	Precoz-R-Root-B2
1-2	8,690	8,876	8,801	8,993
2-3	5,326	5,289	5,110	5,080
3-4	3,668	3,757	3,531	3,665
4-5	2,679	2,710	2,516	2,616
5-6	2,026	2,065	1,969	2,042
6-7	1,625	1,631	1,628	1,700
7-8	1,351	1,383	1,287	1,355
8-9	1,091	1,049	1,104	1,100
9-10	893	919	865	973
10-20	4,564	4,582	4,455	4,631
20-30	1,737	1,775	1,684	1,739
30-40	872	861	869	931
40-50	557	557	519	485
50-60	373	377	360	372
60-70	261	269	247	274
70-80	215	216	203	204
80-90	138	148	164	174
90-100	139	139	144	130
≥100	1,037	1,028	965	955
